# Risk of persistent high-grade squamous intraepithelial lesion after electrosurgical excisional treatment with positive margins: a meta-analysis

**DOI:** 10.1590/S1516-31802012000200009

**Published:** 2012-04-03

**Authors:** Caroline Alves de Oliveira, Fábio Bastos Russomano, Saint Clair dos Santos Gomes, Flávia de Miranda Corrêa

**Affiliations:** I MD, MSc. Obstetrician and Gynecologist, Hospital Federal de Bonsucesso, Bonsucesso, Rio de Janeiro, Brazil.; II MD, PhD. Deputy Director, Department of Education, and Head of the Colposcopy Sector, Instituto Fernandes Figueira (IFF), Fundação Oswaldo Cruz (Fiocruz), Flamengo, Rio de Janeiro, Brazil.; III PhD. Assistant Researcher, Clinical Research Unit, Department of Neonatology. Instituto Fernandes Figueira (IFF), Fundação Osvaldo Cruz (Fiocruz), Flamengo, Rio de Janeiro, Brazil.; IV MD, MSc. Senior Analyst, Cancer Control Program, Divisão de Apoio à Rede de Atenção Oncológica (DARAO), Instituto Nacional do Câncer (INCA), Rio de Janeiro, Brazil.

**Keywords:** Cervical intraepithelial neoplasia, Recurrence, Prognosis, Electrosurgery, Meta-analysis [publication type], Neoplasia intra-epitelial cervical, Recidiva, Prognóstico, Eletrocirurgia, Metanálise

## Abstract

**CONTEXT AND OBJECTIVE:**

Even if precursor lesions of cervical cancer are properly treated, there is a risk of persistence or recurrence. The aim here was to quantify the risks of persistence of high-grade intraepithelial squamous lesions, one and two years after cervical electrosurgical excisional treatment with positive margins.

**DESIGN AND SETTING:**

Systematic review of the literature and meta-analysis at Instituto Fernandes Figueira.

**METHODS:**

This meta-analysis was on studies published between January 1989 and July 2009 that were identified in Medline, Scopus, Embase, Cochrane, SciELO, Lilacs, Adolec, Medcarib, Paho, Wholis, Popline, ISI Web of Science and Sigle. Articles were selected if they were cohort studies on electrosurgical excisional treatment of high-grade squamous intraepithelial lesions with a minimum follow-up of one year, a histopathological outcome of persistence of these lesions and a small risk of bias.

**RESULTS:**

The search identified 7,066 articles and another 21 in the reference lists of these papers. After applying the selection and exclusion criteria, only four articles were found to have extractable data. The risk of persistence of high-grade intraepithelial lesions after one year was 11.36 times greater (95% confidence interval, CI: 5.529-23.379, P < 0.0001) in patients with positive margins and after two years, was four times greater (95% CI: 0.996-16.164), although without statistical significance.

**CONCLUSION:**

This meta-analysis confirms the importance of positive margins as an indicator of incomplete treatment after the first year of follow-up and highlights the need for appropriately chosen electrosurgical techniques based on disease location and extent, with close surveillance of these patients.

## RESUMO

**CONTEXTO E OBJETIVO:**

As lesões precursoras do câncer de colo uterino, mesmo se tratadas adequadamente, têm risco de persistirem ou recidivarem. O objetivo foi quantificar o risco de persistência da lesão intraepitelial escamosa de alto grau (HSIL) em um e dois anos após tratamento excisional eletrocirúrgico do colo uterino com margens comprometidas.

**TIPO DE ESTUDO E LOCAL:**

Revisão sistemática da literatura e metanálise no Instituto Fernandes Figueira.

**METÓDO:**

Metanálise de estudos publicados entre janeiro de 1989 e julho de 2009 identificados em Medline, Scopus, Embase, Cochrane, SciELO, Lilacs, Adolec, Medcarib, Paho, Wholis, Popline, Isis Web of Science e Sigle. Os artigos eram selecionados se fossem estudos tipo coorte sobre tratamento excisional eletrocirúrgico de HSIL com acompanhamento mínimo de um ano e tivessem como desfecho histopatológico a persistência de HSIL com pequeno risco de viés.

**RESULTADOS:**

Foram identificados 7.066 artigos e mais 21 nas listas de referências desses artigos. Após aplicação de critérios de seleção e de exclusão, somente quatro artigos ofereciam dados passíveis de extração. O risco de persistência da HSIL em um ano foi 11.36 vezes maior nas pacientes com margens comprometidas (intervalo de confiança, IC 95%: 5.529-23.379; P < 0,0001) e, em dois anos, chegou a quatro vezes, embora sem significância estatística (IC 95% 0.996-16.164).

**CONCLUSÃO:**

Esta metanálise confirma a importância de margem comprometida como indicador de tratamento incompleto no primeiro ano e ressalta a necessidade de uma adequada escolha da técnica eletrocirúrgica em função da localização e extensão da doença e um acompanhamento adequado dessas pacientes.

## INTRODUCTION

High-grade squamous intraepithelial lesions (HSIL) are considered to be precursors of uterine cervical cancer, and even if adequately treated, they carry a risk of persistence or recurrence.^1^ Some factors highlighted in literature may increase this risk, especially in cases with positive surgical margins.^2^^,^^3^^,^^4^^,^^5^ However, the magnitude of the risk of persistent disease with positive surgical margins is a matter for debate.

Determining whether positive margins in a lesion are really a relevant predictor of persistence of the precursor disease of uterine cervical cancer may aid in managing these patients and thus in guiding new studies. From this, more effective therapeutic techniques and differentiated strategies for follow-up treatment for women at greater risk of residual lesions might be derived.

To shed light on this issue, Ghaem-Maghami et al.^6^ conducted a meta-analysis in which they described the findings among women who were treated using electrosurgical, laser or cold knife excisional procedures. The studies included made it possible to consider that presence of low-grade intraepithelial lesions was an indicator for treatment and represented an outcome of persistence or recurrence. We did not find any other meta-analysis that included the risk of persistence relating only to electrosurgical procedures and with consistent diagnostic criteria of residual precursor disease.

## OBJECTIVE

Our study aimed to evaluate the risk of residual disease after using the electrosurgical excision method, because this method is used preferentially nowadays for ectocervical lesions in our environment, as well as increasingly for endocervical lesions.^7^

We chose to differentiate between residual and recurrent disease and studied the first, since we believe that it is more plausible for lesions to persist after accomplishment of what can be considered to be incomplete excision. Persistence of the lesion was considered to be an outcome when diagnosed within two years after the treatment, as described by van Hamont et al.^8^ All residual lesions were diagnosed by means of a biopsy. To this end, we conducted a systematic review of the literature and a meta-analysis in order to quantify the risk of persistence of HSIL with positive margins, one and two years after electrosurgical excisional treatment on the uterine cervix.

## METHODS

### Identification of studies

We conducted a systematic review of the literature and a meta-analysis, and followed the description known as preferred reporting items for systematic reviews and meta-analyses (PRISMA).^9^ At the end of the study, in order to ensure quality in the description of the meta-analysis, we used the checklist for meta-analyses on observational studies in epidemiology (MOOSE): a proposal for reporting studies.^10^

Our search sources were electronic means (Medline, Scopus, Cochrane, Lilacs, SciELO, Embase, Popline, Adolec, Medcarib, ISI Web of Science, Wholis, Paho and Sigle), reference lists of reputable articles in master’s degree and PhD thesis databases (Coordenação de Aperfeiçoamento de Pessoal de Nível Superior [Capes], Fundação Oswaldo Cruz [Fiocruz] and Universidade de São Paulo [USP]) as well as contacts with authors within the field for possible searches for relevant material not yet published.

**Chart 1.**
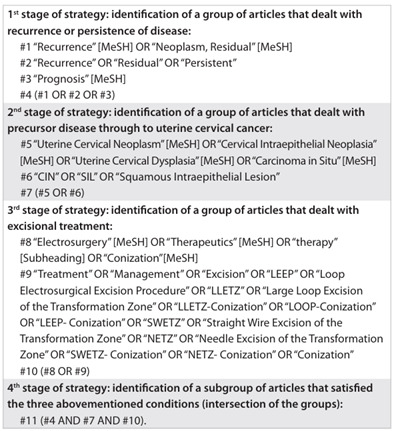
Description of initial strategy for identifying topic-related articles on Medline

The initial strategy using Medline sought to correlate three groups of articles with keywords relating to: (1) recurrence or persistence; (2) precursor disease for uterine cervical cancer; and (3) excisional treatment. Thus, the MeSH (Medical Subject Heading) terms and other terms that the authors could use were listed as shown in Chart 1.

The following limits were applied: NOT “Breast Neoplasm” [MeSH] NOT “Neoplasm Invasiveness” [MeSH] NOT “BCG Vaccine” [MeSH] NOT “Urinary Bladder Neoplasm” [MeSH] NOT “Laryngeal Neoplasm” [MeSH] NOT “Vulvar Neoplasm” [MeSH] NOT “Lymphatic Metastasis” [MeSH] NOT “Lung Diseases” [MeSH] NOT “Sarcoidosis” [MeSH] NOT “Ovarian Neoplasm” [MeSH] NOT “Lung Neoplasm” [MeSH] NOT “Skin Neoplasm” [MeSH] NOT “Melanoma” [MeSH] NOT” Stomach Neoplasm” [MeSH] NOT “Anus Neoplasm” [MeSH] NOT “Thyroid Neoplasm” [MeSH] NOT “Lymphoma” [MeSH].

The search was limited to articles published between 1989 and July 2009 that were qualified as original articles (published papers classified in Medline as clinical trial, randomized controlled trial, comparative study, controlled clinical trial, journal article or multicenter study). There were no language constraints.

### Selection of studies

Articles were selected if they were cohorts on electrosurgical excisional treatment of HSIL with a minimum follow-up of one year^11^^,^^12^ and a histopathological outcome of HSIL persistence.^13^

The eligibility of the studies was evaluated blindly with regard to authors, journals and funding sources by two researchers. There was no disagreement among researchers. Studies were considered eligible if they did not present a risk of selection bias (i.e. they used a well-defined and representative sample of the patient population), if the sample loss was less than 20% and if there were similar follow-ups among the independent comparisons of margin status.^14^

### Data extraction

The results from the studies were extracted if the outcome was evaluated after one or two years of follow-up. The objective was to extract the following data: sample size, electrosurgical technique described, number of patients with a new diagnosis after one or two years of free or positive surgical margins, and other information that might improve the analysis on the results (via colposcopy, electrosurgical technique and histopathological descriptions).

### Statistical analysis

In order to evaluate clinical and methodological heterogeneity, we described the methodology, participants’ characteristics and intervention type and defined the outcomes from eligible articles. Statistical heterogeneity was evaluated by means of the chi-square test.

The meta-analysis was performed using the Stata 10.1 software, by means of the fixed-effect model,^15^ and the results were expressed as relative risks with 95% confidence intervals and absolute risks.

Since this was a systematic review (i.e. using a method in which scientific publications are the subject of investigation, without relating to patients), there was no need for approval of the present study from the ethics committee, for it to be implemented.

## RESULTS

**Figure 1.**
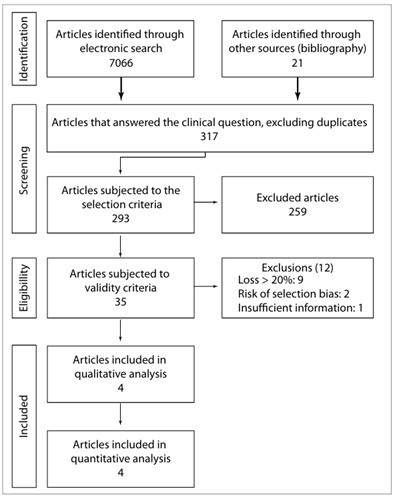
Flowchart for data search, with inclusion and exclusion of articles

We identified 7,066 articles using similar strategies in each electronic source. Their titles were analyzed, and 524 articles relating to the topic were retained. Among these, 227 were duplicates, thus leaving 297 for a more detailed analysis of titles and abstracts. A further 21 articles were extracted through manual searches in the reference lists of these 297 articles. One article was a duplicate, thus producing a total of 317 articles. Twenty-four were excluded because they were reviews, replies to authors of published reviews or in languages that we were unable to translate into Portuguese (seven articles in the following languages: Bulgarian, Chinese, Finnish, Hebrew, Russian and Serbian). Therefore, 293 articles were subjected to the selection criteria. Out of these, only 35 went forward for eligibility evaluation. Twenty-three of these were considered to be eligible for extraction, but only four contained extractable data that could contribute towards conducting a meta-analysis (Figure 1). The reasons for the exclusions with regard to the impossibility of data extraction are listed below (with numbers of articles):

Insufficient data (4); no separation between electrosurgical technique or cold knife conization (1); no differentiation of compromised margins of positive endocervical curettage as a prognostic factor (1); impossible to separate lesion grade data regarding margins with recurrence, i.e. low or high-grade intraepithelial lesions (1); no data on the time of diagnosis to allow differentiation between residual and recurrent disease (5); no data to confirm histology of recurrence (2); no separate data relating to HSIL (4); and margins considered to be compromised by human papillomavirus (HPV) infection alone (1).

We attempted to contact the 33 authors from whom we believed that we needed to obtain more information about their articles, by sending e-mails or letters to the addresses identified in their papers. However, up to the time of final writing of the present study, we had not obtained any response.

**Table 1.** Risk of residual disease one year after electrosurgery excisional treatment with positive margins

Study	Relative risk	95% confidence interval	% weighting in the final result
Chang et al.^16^	10.000	4.632-21.588	90.03
Goya-Canino et al.^17^	23.727	2.715-207.375	9.97
Summary measurement	11.369	5.529-23.379	100.00

P = 0.459 (chi-square test for heterogeneity); I-squared (variation of relative risk relating to heterogeneity) = 0.0%; relative risk test = 1; z = 6.61; P < 0.0001.

**Table 2.** Risk of residual disease two years after electrosurgery excisional treatment with positive margins

Study	Relative risk	95% confidence interval	% weighting in the final result
Gardeil et al.^18^	10.588	0.578-193.861	22.80
Verguts et al.^19^	2.071	0.421-10.197	77.20
Summary measurement	4.013	0.996-16.164	100.00

P = 0.297 (chi-square test for heterogeneity); I-squared (variation of relative risk relating to heterogeneity) = 8.2%; relative risk test = 1; z = 1.96; P = 0.051.

**Figure 2.**
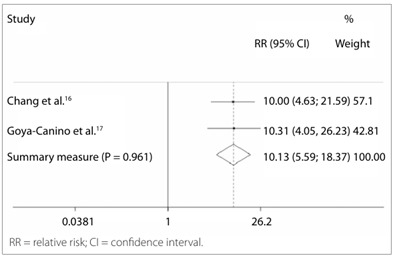
Forest plot including the two studies that analyzed the risk of residual disease one year after electrosurgical excisional treatment with positive margins.

**Figure 3.**
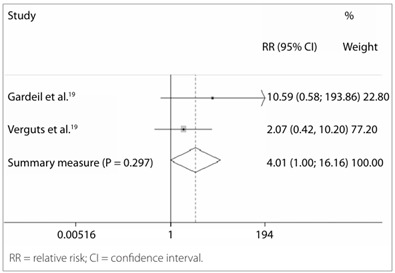
Forest plot including the two studies that analyzed the risk of residual disease two years after electrosurgical excisional treatment with positive margins.

The following studies contributed towards risk estimation after one year:

- Chang et al.^16^: 172 patients who underwent conization followed by hysterectomy, independent of the results relating to surgical margins, at the National University Hospital of Taiwan. Our review only used the information from 129 cases of high-grade intraepithelial lesions. Sixteen patients had residual disease, among 24 with positive margins, while seven had residual disease among 105 with negative margins.
- Goya-Canino et al.^17^: 382 patients who were followed up through appointments at the cervical pathology clinic of the Mother-Child University Hospital of Canary, Spain. Among these, 305 underwent loopconization to treat high-grade intraepithelial squamous lesion (cancer cases had been excluded), and all of them underwent cyto-colposcopic follow-up. Four patients had residual disease among 44 with positive margins, while one had residual disease among 261 with negative margins.

The following studies contributed towards risk estimation after two years:

- Gardeil et al.^18^: 583 patients who had been referred from other hospitals, family doctors and family planning units, to Coombe Women’s Hospital in Dublin, Ireland. We only took into consideration the data on 225 patients who had a histopathological diagnosis of HSIL (NIC III) and had undergone LLETZ (large-loop excision of the transformation zone) by means of a diathermal loop of depths ranging from 10 mm to 20 mm. There was no information on how many patients underwent conization and how many underwent only excision of the transformation zone. The follow-up was cyto-colposcopic. There was a loss of 42 patients. Out of the remaining 183 patients, four had residual disease among 84 with positive margins, while no patient had residual disease among 99 with negative margins.
- Verguts et al.^19^: 72 women with high-grade intraepithelial squamous lesions who were treated by means of LLETZ in the Department of Gynecology of Gasthuisberg University Hospital in Leuven, Belgium, with cyto-colposcopic follow-up. Two patients had residual disease among 14 with positive margins, while four had residual disease among 58 with negative margins.

The results extracted from the four selected articles were set up in an electronic spreadsheet, which was processed using the Stata 10.1 software for meta-analysis.

The risk of residual disease one year after electrosurgical treatment was 11.369 times greater among the patients with positive margins, as can be seen in Table 1 and Figure 2. The absolute risk of presenting residual disease one year after a diagnosis of positive margins reached 29.4%, versus 2.1% in the cases with free margins.

The risk of residual disease two years after electrosurgical treatment was four times greater among the patients with positive margins, but without reaching statistical significance (Table 2 and Figure 3). The absolute risk of presenting residual disease two years after a diagnosis of positive margins was 6%, versus 2.5% in the cases with free margins.

## DISCUSSION

We observed that there was a notable risk of residual disease one year after electrosurgical treatment in patients with positive margins. The estimated risk was about 11 times higher than among patients with free margins. The confidence intervals show that this risk can be estimated as at least 5.5 times higher and at most 23.3 times higher. The absolute risk of presenting residual disease over the first year after a diagnosis of positive margins reached 29.4%, versus 2.1% in the cases with free margins. The risk calculation after two years showed that the estimated risk was about four times greater than among patients with free margins, although this did not reach statistical significance.

This association is more important with regard to residual disease over the first year of follow-up, which reinforces the premise that this factor may be more connected to incomplete treatment than to recurrent disease.

The studies that were evaluated showed notable variation in the percentage of positive margins. In Gardeil et al.,^18^ almost half of the sample had positive margins (45%) and 4.7% presented residual disease. On the other hand, in Verguts et al.,^19^ 19% had positive margins and 2.77% had residual disease. In the 1997 study,^18^ it was reported that only seven cases presented thermal artifacts that hindered margin evaluation. The percentage of positive margins of 45% is extremely high and allows us to infer that it is possible that some cases may have been misevaluated with regard to choosing the excisional method or with regard to the margins.

None of the studies mentioned any well-defined diagnostic criteria for positive margins. However, it is known that at this phase, there are problems that might also have influenced the variation of percentages of positive margins observed among the studies. Although none of the studies mentioned the anatomical site of the positive margin (endocervical or ectocervical), it is important to report which margin is compromised, in order to provide better follow-up for patients with endocervical margin involvement. In this latter type of case, it may be more difficult to diagnose a residual lesion.

Chang et al.^16^ gave a detailed description of how the specimen was processed. They reported that the specimen was cut at 12 o’clock, parallel to the axis of the cervical canal, fixed onto a cork plate and left in formaldehyde until the next day. Following this, the specimen was cut every 3-4 mm and then received perpendicular cuts to the surface of the mucous membrane. However, Gardeil et al.^18^ only reported that the specimens were opened at 12 o ’ clock and put in formaldehyde. The other articles did not mention the specimen processing.

All the problems relating to the histopathological analysis (diagnostic criteria for positive margins and thermal damage) and to the surgical technique (surgical excision of more than one segment and specimen processing) might explain the discrepancy in the results relating to positive margins and residual disease.

After performing this meta-analysis, we checked the list of the 26 articles included in the treatment type “electrosurgery” in the study by Ghaem-Maghami et al.^6^ (compared with the four articles included in our study). We noted that sixteen articles included in that study had been excluded in the selection phase of our work. Moreover, one article on cold conization^20^ was wrongly included in the follow-up group of studies after electrosurgery. Among the remaining ten articles, two were excluded in the validity evaluation. Six were excluded in our study at the data extraction phase and thus, out of the initial list of 26 articles included in the meta-analysis of Ghaem-Maghami et al.,^6^ only two^18^^,^^19^ were considered suitable for inclusion in our meta-analysis.

It needs to be highlighted that the risk shown in the meta-analysis by Ghaem-Maghami et al.^6^ was almost 50% lower (RR 6.09) than what we observed in our work. Ghaem-Maghami et al.^6^ collected data from articles reporting all therapeutic methods (cold conization, laser and electrosurgery). The lower association that they found between the studied factor and the outcome may be translated into difficulty in diagnosing positive margins, especially in cases undergoing laser excision, and may lead to underestimated risk. However, when calculating the risk in articles on electrosurgery alone, Ghaem-Maghami et al.^6^ took both high and low-grade lesions together as the outcome, thereby reaching a risk rate that was approximately one third of what we observed in our work (RR 3.34). This may have been due to inclusion of articles with lower levels of validity and thus greater likelihood of bias. Another possibility is that by including low-grade lesions within the outcome, their greater frequency may lead to a lower difference in incidence between the comparison groups, thus not representing the residual disease properly.

Another issue to be addressed is that the meta-analysis by Ghaem-Maghami et al.^6^ used Medline as the only data source. Not only are the other research databases mentioned earlier all of importance, but also one article included in our meta-analysis^17^ was only retrieved from Scopus in the Spanish language. This article was not considered in the meta-analysis by Ghaem-Maghami et al.,^6^ and it made an important contribution towards our results, since it achieved a relative risk of ten, thereby increasing the value of our summary measurement.

The four studies included in our meta-analysis rigorously met all the prerequisites for the issue addressed. Nonetheless, the meta-analysis may give a false impression that the results have been established with a high degree of precision, when in reality the main results depend on many premises that have yet to be supported.^21^

In one of the studies included, Gardeil et al.^18^ provided data on patients who were reviewed at 6 and 24 months, but not one year. Thus, absence of a simple piece of information meant that we were prevented from using this study to contribute towards the cutoff point.

On several occasions in the studies included, the data described in the text differed from what was presented in the tables. At times, the information regarding procedures was vague. Another limitation of the present study was that we did not have access to the database of each article. Hence, the calculated data was not adjusted, and the possibility of confounding thus cannot be dismissed.

We aimed to study the risk of residual disease separately for excision of the transformational zone and electrosurgical conization. However, all the studies included related to conization or did not present any differentiation regarding the technique applied. Further studies should be conducted, with better description of the surgical technique applied, in order to establish whether there is any difference in the risk. Moreover, we suggest that objective standardized criteria should be defined for the diagnosis of positive margins.

Another limitation of our work was that it was impossible for us to translate into Portuguese seven articles in the following languages: Bulgarian, Chinese, Finnish, Hebrew, Russian and Serbian.

Despite these limitations, after conducting a systematic review and then a meta-analysis with stringent methodological testing that is repeatable, we achieved a significant result in which the estimated risk of detecting residual disease one year after electrosurgery was approximately eleven times greater when there are positive margins.

Thus, we may assert that there is significantly higher likelihood that the disease will persist during the first year, in cases in which the surgical specimen shows positive margins, and accordingly, these patients should have a differentiated follow-up. We highlight the importance of choosing an appropriate electrosurgical technique, with due regard to the location and extent of the lesion, in order to reduce the risk of incomplete treatment of pre-invasive cervical lesions.

Further studies should be conducted to make it clear what the best possible follow-up for such patients would be.

## CONCLUSIONS

The risk of residual disease one year after electrosurgical excisional treatment with positive margins is about 11 times greater than among patients with free margins. The risk of residual disease two years after such treatment is four times greater, but without statistical significance.

The absolute risk of presentation of residual lesions over the first year is 29.4% among patients whose specimen showed positive margins whereas over the second year, it is 6%.

Attention is required regarding proper indications, appropriate surgical procedures, correct processing for the excised specimen and appropriate choice of technique, which needs to be individualized for each case, in order to reduce the risk of residual disease. Despite the small number of studies included in this meta-analysis and the limitations mentioned above, this study clearly shows the importance of the risk of treatment failure when there are reports of positive margins. Further studies should be conducted to determine the best strategy for following up these patients, especially during the first year after treatment.
